# Correlations of obstetric anal sphincter injury (OASIS) grade, specific symptoms of anal incontinence, and measurements by endoanal and transperineal ultrasound

**DOI:** 10.1007/s40477-020-00485-4

**Published:** 2020-05-31

**Authors:** Claes Ignell, Ann-Kristin Örnö, Andrea Stuart

**Affiliations:** 1 Department of Obstetrics and Gynecology, Helsingborg Hospital, Fredrik Cöstersgata 6, 254 43 Helsingborg, Sweden; 2 Department of Clinical Sciences, Lund University, Malmö, Sweden; 3 Department of Obstetrics and Gynecology, Sahlgrenska University Hospital, Göteborg, Sweden

**Keywords:** Endoanal ultrasound, Transperineal ultrasound, Obstetrical anal sphincter injury, Wexner score

## Abstract

**Purpose:**

The aim of the study was to investigate the association between the initial grade of obstetrical anal sphincter injury (OASIS), and Wexner score parameters, with ultrasonographic findings by endoanal ultrasound (EAUS, golden standard) and transperineal ultrasound (TPUS) 6 months post-partum.

**Methods:**

Fifty-nine women after primary repair of OASIS were included at Helsingborg Hospital, Sweden, 2016–2017. Six months post-partum the women filled in a questionnaire regarding symptoms of anal incontinence by the Wexner score and were scanned with EAUS and TPUS (resting state and contracting state) for classification of the residual defect by a modified Starck score.

**Results:**

Correlations were found between the OASIS grade and residual defects; length (*r*_s_ = 0.41, *P* = 0.003), depth (*r*_s_ = 0.38, *P* = 0.006) and angle (*r*_s_ = 0.40, *P* = 0.004) of the external anal sphincter (EAS) measured with TPUS in resting state. Using EAUS, correlation between OASIS grade and EAS depth (*r*_s_ = 0.35, *P* = 0.007) and angle (*r*_s_ = 0.37, *P* = 0.004) were similar, but there was no correlation with length (*r*_s_ = 0.20, *P* = 0.14). Between incontinence to gas and the angle of the residual defect in the IAS using TPUS in resting state, correlation was moderate (*r*_s_ = 0.42, *P* = 0.003). Regarding incontinence to liquid stool, measurements by TPUS in resting state of EAS residual defect depth (*r*_s_ = 0.46, *P* < 0.001) and angle (*r*_s_ = 0.44, *P* = 0.001) also correlated moderately. Both corresponding correlations using EAUS were weaker.

**Conclusion:**

Defects measured with EAUS and TPUS six months post-partum correlated to initial OASIS grade and symptoms of anal incontinence. Specific symptoms correlated with specific anatomical defects, and TPUS was not an inferior method to EAUS.

## Introduction

Obstetric injuries to the anal sphincter (OASIS) complex affect approximately 5% of women in conjunction with childbirth [1–4], with the main risk factors being nulliparity and operative vaginal delivery [2]. OASIS are a main cause of anal incontinence, and can also lead dyspareunia, fecal urgency, perineal pain [5, 6] and is in addition associated with sexual embarrassment and psychological effects [7].

The anal sphincter complex constitutes of the internal anal sphincter (IAS), consisting of smooth muscle and the external anal sphincter (EAS), consisting of skeletal muscle. Obstetrical injuries to the anal sphincter are classified as proposed by Sultan [8].

One might assume that the larger the injury, the more symptoms of anal incontinence should be evident. However, data is inconsistent regarding both the correlation between endosonographically defined defects and of, but also the initial grade of defect and symptoms of fecal incontinence [9–11].

Endoanal ultrasound (EAUS) is considered gold standard in ultrasonographic assessment of OASIS. Other ultrasound modalities are on the rise, recently three-dimensional transperineal ultrasound (TPUS) was presented as a screening tool in assessment of residual defects after OASIS [12], We have previously showed a strong a correlation between EAUS and TPUS in the assessment of residual defects six months after primary suturing of OASIS [13], the TPUS score showed a strong correlation with EAUS score during both pelvic floor relaxation and contraction (*r*_s_ = 0.74, *P* < 0.001 and *r*_s_ = 0.77, *P* < 0.001, respectively). Significant correlations between EAUS and TPUS were found when assessing the depth, length and angle of the defect, regarding both the EAS and IAS. The strongest correlation between EAUS and TPUS was regarding the depth of the injury (*r*_s_ = 0.71, *P* < 0.001).

The aim with the current study was to investigate the association between the initial grade of OASIS in conjunction with labor with the ultrasonographic findings with both EAUS and TPUS six months post-partum. Secondly, we aimed to assess the correlation between specific ultrasound findings with both EAUS and TPUS and each separate variable in the Wexner score.

## Material and methods

Fifty-nine women after primary repair of OASIS were included at Helsingborg Hospital, Sweden, 2016–2017. Our hospital has standardized clinical guidelines when repairing OASIS. The primary repair was performed by a specialist in obstetrics and gynecology or by a resident physician under the supervision of a specialist. The anorectal mucosa was sutured with Vicryl 3-0 or 4-0 running sutures, the EAS with Vicryl 2-0, and IAS with Vicryl 3-0, interrupted sutures. At the discretion of the surgeon, the end-to end suturing technique or overlapping technique was chosen regarding the EAS. Prophylactic antibiotics are recommended to all grade 3–4 injuries. No patients in the study required secondary repair of the primary injury.

The study group and method are the same as in a previously published article [13]. All women with OASIS in conjunction with delivery were offered a follow up appointment 6 months after delivery. All women showing up at the follow up appointment were asked to participate in the study. Two patients declined to participate for unspecified reasons. No information exists regarding which patients did not show up at the follow up appointment. Exclusion criterion was the inability to understand and read Swedish. After written consent, all patients went through a standardized interview using a written questionnaire, clinical examination and ultrasound examination by one of the authors (AÖ). All women were asked for symptoms of anal incontinence and filled in a form regarding symptoms according to the Wexner score [14], giving points for liquid and solid fecal incontinence, gas incontinence, and lifestyle alteration and wearing a pad [12]. The score ranges from 0 to 20, with 20 being total incontinence.

The ultrasound investigation took place in the dorsal lithotomy position with endoanal ultrasound and transperineal ultrasound. Both ultrasound modalities were performed by the same author during one clinical appointment. The endoanal ultrasound (BK, Medical Ultrasound scanner 1202, bandwidth 4–12 MHz) was considered gold standard, and produces a 360° cross-sectional view. Endoanal ultrasound is performed only with the patient resting the pelvic floor. Voluson GE E8 was used with a 3D/4D convex transducer (RAB4-8-D), (bandwidth 8–12 MHz) for transperineal ultrasound.

For assessment of the sphincter complex the convex transducer was positioned over the perineum in a horizontal position, and angled caudally towards the anal sphincter. The ultrasound assessment, henceforth defined as TPUS, was firstly performed in resting state, and secondly in contracting state while the woman was doing maximal contraction of the anal complex and thirdly while the patient was doing the Valsalva maneuver.

**Fig. 1:**
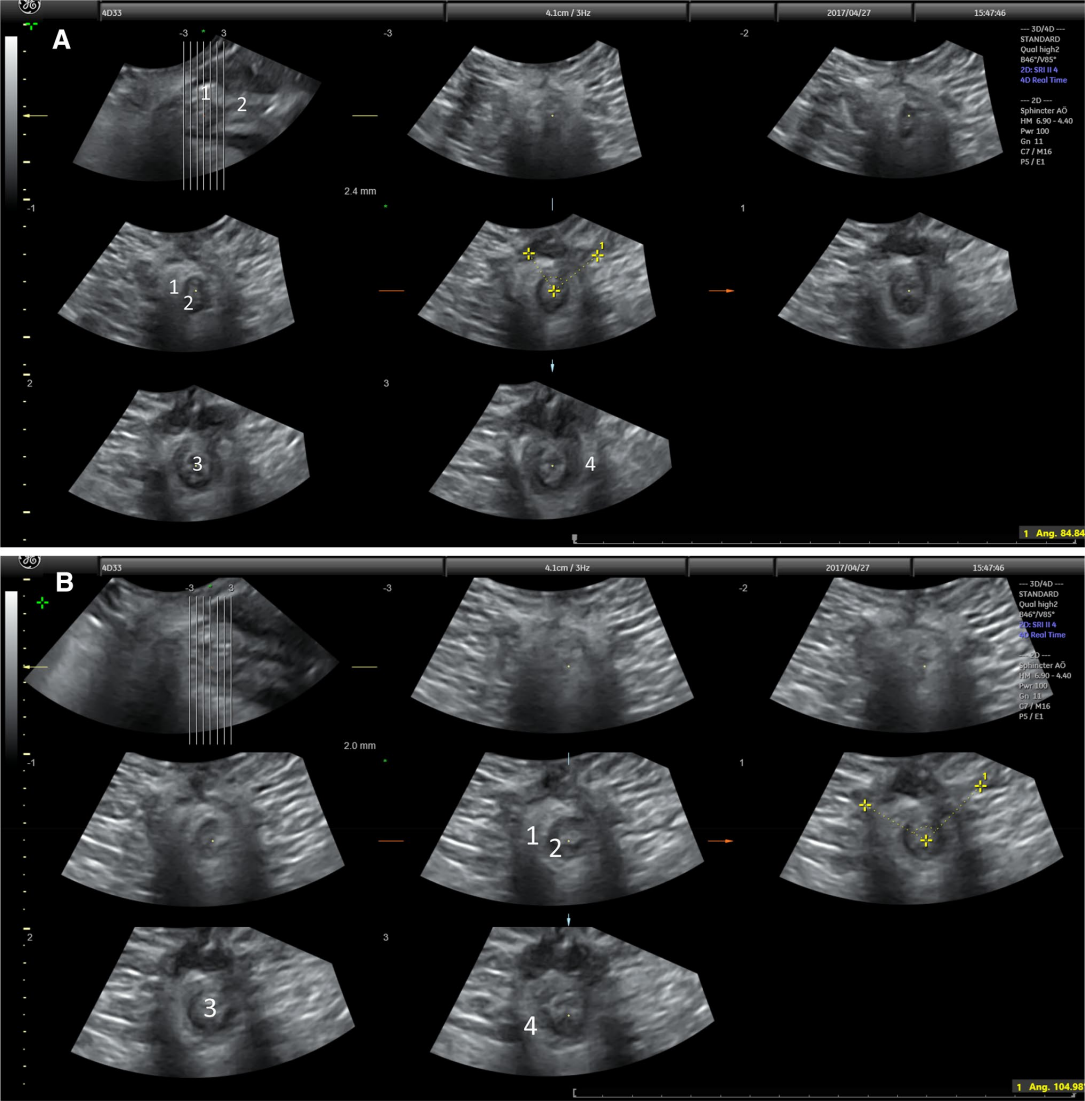
Transperineal scan with the 4D/3D probe held in a transverse position related to the perineum. **a** A tomographic transversal scan of the anal canal at rest. A partial defect in the EAS remains after the repair. **b** The canal during a during a voluntary contraction. The angle between the torn ends are increased in **b**, 104° compared to 84° in resting. EAS (1) IAS (2), lumen of the anal canal (3), levator ani (4)

Figure 1a shows a transversal scan of the anal canal at rest with 3D/4D ultrasound. Figure 1b shows the anal canal during a voluntary contraction. A voluntary squeeze of the pelvic floor results in a cranial/inward movement of the anal canal. Therefore, difficulties exist measuring the exact same point in resting and during a squeeze. The 4D/3D system is essential giving possibility to correct the position of the tomographic sequences and correct any deviation from the midline that could occur during the squeeze. At the anal verge there is a detectable decrease in the diameter around the caudal/ distal part of the IAS during a squeeze. A tear in the IAS and the EAS will not decrease the cranial movements of the anal canal during a squeeze, but instead an increase in the angle between the torn ends of the sphincter muscle will be seen. The increased angle may represent a true defect as opposed to a scar where no such increase can be seen during a squeeze.

All scans were analyzed off-line, at a different point of time than the clinical examination, by two examiners (AS and AÖ). The patients’ personal identification numbers were exchanged with an arbitrary number used as an identification key. Therefore, the examiners were blinded to the patients’ symptoms and identity. Firstly, both examiners categorized the defects in the anal sphincter complex separately, then their results were compared. In the case of a disagreement regarding the categorization of the ultrasound assessment, reanalysis and consensus was reached. Only the common score was documented. Tears in both the EAS and IAS were categorized according to a modified scale by Starck et al. [15] analyzing the length, depth, and radial extent, as described previously [14], and shown in Table 1. Partial defects of the EAS involving less than 50% of the thickness or length gave 0 points in our scoring system (Table 1). Only full thickness defects or involving 50% or more of the IAS gave 1 point.

**Table 1:** Modified ultrasound score according to Starck et al. (maximum score 8)

	Score	0	1	2	3
External anal sphincter	Length	< 50%	> 50%	–	–
	Depth, radial extent	< 50%	> 50%	100% depth, < 90°	100% depth, > 90°
Internal anal sphincter	Length	< 50%	> 50%	–	–
	Depth, radial extent		100% depth, < 90°	100% depth, 90°–180°	100% depth, > 180°

Using tomographic ultrasound imaging (TUI), parallel slices (2.6 mm) were obtained with TPUS, with the caudal slice at the subcutaneous part of the EAS and the cranial slice at the puborectal muscle. Moving the TUI along the anal canal helped to estimate the beginning and end of the tear. A sphincter defect was defined as a discontinuity in the endosonographic appearance of the internal or external anal sphincter. The assessment of defects in the EAS was analyzed from the most distal part of the anal complex, defined as when both the IAS and EAS could be seen simultaneously on ultrasound. The most cranial/proximal extension of the IAS and EAS were assessed 5 mm distally to the puborectal muscle, when measuring the length of the injury. Scar tissue is seen as a hyperechoic irregular, without distinct structures. A true defect is usually hypoechoic. Also, as described, when the muscle contracts, the angle will increase in the case of a defect.

When the depth of the defect more than 50% was adjacent to a defect with a depth less than 50%, the radial extent of the two defects were assessed together. When the IAS and EAS were not assessable due to poor quality of the off-line ultrasound image, they were excluded for further analysis, and marked as “non-assessable” [14].

### Ethical approval

The ethics committee of Lund University, Sweden (reference number 2009/15) approved the study, and written informed consent was obtained from all participants.

### Statistical analysis

Statistical analysis was performed with use of the IBM SPSS Statistics 24 (IBM Corporation, Armonk, NY). The Spearman correlation coefficient (*r*_s_) was used to calculated the correlation between the original grade of OASIS and each parameter within the ultrasound score (length, depth, angle) for both the EAS and IAS and also to calculate the correlation between each parameter in the Wexner score and the initial grade of OASIS after delivery. A *r*_s_ of 0–0.19 was considered as “very weak”, 0.20–0.39 as “weak” and 0.40–0.59 as “moderate”, 0.60–0.79 as “strong”, and 0.80–1.0 as “very strong”. Results were considered statistically significant when *P* < 0.05 (2-sided).

## Results

Descriptive characteristics of the study population are shown in Table 2.

**Table 2:** Descriptives of included women by clinical classification of OASIS grade after delivery

	OASIS grade			
	3a (*n* = 22)	3b (*n* = 17)	3c (*n* = 12)	4 (*n* = 6)
Age (years)	31 (28–32)	32 (27–33)	28 (26–31)	31 (24–34)
BMI (kg/m^2^)	25 (23–28)	24 (22–29)	22 (20–24)	24 (22–28)
Primipara	17 (77)	11 (65)	9 (75)	5 (83)
Previous cesarean	2 (9)	3 (18)	2 (17)	–
Spontaneous labor	18 (82)	14 (82)	11 (92)	5 (83)
Epidural	13 (59)	9 (53)	6 (50)	5 (83)
Inf. Oxytocin	16 (73)	11 (65)	7 (58)	4 (67)
Episiotomy	1 (5)	3 (18)	2 (17)	–
Fundal pressure	2 (9)	1 (6)	1 (8)	–
Instrumental delivery	7 (32)	4 (24)	2 (17)	3 (50)
Asphyxia	4 (18)	2 (12)	1 (8)	–
Birth weight (g)	3755 (3330–4230)	3715 (3545–3945)	3713 (3470–4003)	3655 (3460–3690)

Dichotomous variables, *n* (%); Other variables, median (lower–upper quartile limits)

*OASIS* obstetric anal sphincter injuries

The correlation between increasing grade of OASIS (grade 3a, 3b, 3c and grade 4) and ultrasound score six months post-partum for both EAUS and TPUS are shown in Table 3. As specified in the table, significant correlations were found between the grade of OASIS and all parameters (depth, length, and angle) of the residual defects measured in both the EAS and IAS when TPUS was performed in contracting state. TPUS in resting state showed similar results, but IAS residual defect in length was not significantly correlated with the original grade of OASIS. When measured with EAUS, a significant correlation was found between the original grade of OASIS and the depth and angle of the defect in the EAS and also the length, depth, and angle of the residual defect in the IAS.

**Table 3:** Correlations of OASIS grade and ultrasound score by EAUS and TPUS (in resting and contracting state, respectively) 6 months post-partum

	EAUS		TPUS, resting		TPUS, contracting	
	*r*_s_	*P*	*r*_s_	*P*	*r*_s_	*P*
EAS						
Length	0.20	0.14	0.41	0.003	0.42	0.003
Depth	0.35	0.007	0.38	0.006	0.34	0.016
Angle	0.37	0.004	0.40	0.004	0.36	0.013
IAS						
Length	0.28	0.038	0.25	0.09	0.40	0.004
Depth	0.38	0.003	0.33	0.020	0.33	0.019
Angle	0.39	0.002	0.32	0.026	0.36	0.009

*EAS* external anal sphincter, *EAUS* endoanal ultrasound, *IAS* internal anal sphincter, *OASIS* obstetric anal sphincter injuries, *r*_*s*_ Spearmans rho, *TPUS* transperineal ultrasound

A significant correlation was found between the total Wexner score at six months postpartum and increasing grade of OASIS (*r*_s_ 0.35, *P* = 0.008). A significant correlation also was found between OASIS grade and the parameter “gas incontinence” (*r*_s_ 0.36, *P* = 0.006) and “liquid stool incontinence” (*r*_s_ 0.29, *P* = 0.031). No significant correlation was found between the parameters “lifestyle alternation” (*r*_s_ 0.02, *P* = 0.87), and “wearing a pad” (*r*_s_ 0.09, *P* = 0.53), and OASIS grade. Only two patients had incontinence to solid stools, nevertheless, the correlation with the Wexner score was close to significant (*r*_s_ 0.25, *P* = 0.056).

The correlations of the specific parameters of gas incontinence and liquid stool incontinence of the Wexner score and the different parameters (length, depth, angle) of ultrasonographic Starck score are presented in Table 4. Regarding gas incontinence, IAS residual defect angle examined by TPUS in resting state had a significant, moderate, correlation. Regarding the parameter “liquid stool incontinence”, TPUS in resting state was denoted by moderate, significant, correlations of the residual defect in the depth and angle of the EAS.

**Table 4:** Correlations of incontinence to gas (a) and incontinence to liquid stool (b), and modified Starck scores by EAUS and TPUS

	EAUS		TPUS, resting		TPUS, contracting	
	*r*_s_	*P*	*r*_s_	*P*	*r*_s_	*P*
a. Incontinence to gas (*n* = 39)						
EAS						
Length	0.29	0.026	0.30	0.031	0.24	0.095
Depth	0.35	0.006	0.25	0.077	0.34	0.016
Angle	0.32	0.014	0.24	0.082	0.34	0.018
IAS						
Length	0.24	0.068	0.21	0.14	0.32	0.021
Depth	0.28	0.030	0.32	0.023	0.36	0.010
Angle	0.29	0.024	0.42	0.003	0.35	0.012
b. Incontinence to liquid stool (*n* = 9)						
EAS						
Length	0.15	0.25	0.19	0.19	0.18	0.21
Depth	0.22	0.099	0.46	< 0.001	0.26	0.070
Angle	0.17	0.20	0.44	0.001	0.35	0.013
IAS						
Length	0.27	0.037	0.32	0.023	0.27	0.052
Depth	0.22	0.099	0.26	0.071	0.29	0.042
Angle	0.23	0.078	0.33	0.019	0.30	0.03

*EAS* external anal sphincter, *EAUS* endoanal ultrasound, *IAS* internal anal sphincter, *r*_*s*_ Spearman’s rho, *TPUS* transperineal ultrasound

## Discussion

Our study, including 59 patients, assessed residual defects 6 months post-partum after primary suturing of OASIS, shows correlations with initial grade of OASIS, and between specific symptoms of fecal incontinence and specific ultrasonographic findings of residual injury. We assessed the residual defects with EAUS, TPUS in resting state and TPUS in contracting state. With all three ultrasound techniques significant weak correlations between the original grades of OASIS were found, most pronounced for TPUS in resting state. Furthermore, specific symptoms of gas and liquid stool incontinence correlated moderately, with a higher significance, with specific anatomical defects in the IAS and EAS, respectively. Measurements with TPUS had similar or stronger correlations between residual defects and symptoms than those of EAUS.

The Wexner score is commonly used to assess symptoms of fecal incontinence after perineal trauma. However, it is composed of both specific parameters of fecal incontinence (gas, liquid and solid stool incontinence), and unspecific parameters of fecal incontinence (lifestyle alternation, wearing a pad). Our results showed significant correlations between the initial grade of OASIS and the specific parameters of fecal incontinence, no correlation between the initial grade of OASIS and the unspecific parameters were found.

Our results showed a moderate significant correlation between gas incontinence and the angle and depth of the residual tear in the IAS, as measured by both EAUS and TPUS in resting and contraction. Residual defects in the EAS measured with TPUS in resting, showed a significant moderate correlation between incontinence to liquid stool and both the angle and length of the residual tear in the EAS. When performing EAUS or TPUS the first projection seen on the ultrasound screen of the anal sphincter is an axial view in which the angle and depth of the tear can be assessed. In order to measure the length of the tear, the ultrasound images must be turned to a sagittal/longitudinal view in the computer program. In our opinion, the angle and depth of the tear are the easiest to see and measure. Our results also support that the depth and angle of the EAS have stronger correlations with symptoms than the length of the defect in the EAS. As all obstetric units do not have access to endoanal ultrasound, it would be of great clinical benefit if an initial evaluation of the sphincter complex could be performed using transperineal ultrasound assessing the depth and angle of the tear.

The results of TPUS in resting state are in line with the physiological function of the IAS and EAS [15].

Endoanal ultrasound is considered gold standard in assessing defects in the anal sphincter complex. Our results, however, showed stronger correlations between measurements by TPUS, in both resting and contraction, with specific symptoms of fecal incontinence, than the correlation of measurements by EAUS. Unexpectedly, our results revealed no correlation between incontinence to liquid stool and residual defects in the EAS measured with gold standard EAUS. Furthermore, TPUS showed correlations of the same magnitude as EAUS with the initial grade of OASIS (grade 3a, b, c, and 4). Our results support further studies to verify the use of TPUS when examining the patient after suturing of OASIS.

A strength of our study is the fact that we could analyze each parameter in the Wexner score separately and performed ultrasound assessments with two clinically available modalities. A limitation of our study is that we could not calculate interpersonal agreement regarding assessment of ultrasound defects, as we only documented the common ultrasound score. Also, as we included both primipara and multipara, symptoms and ultrasound findings could be confounded by advents during the first delivery.

Ramage et al. investigated 177 patients from 3 to 24 months postpartum and found no decrease in anal function with increasing OASIS grades [9]. Roos et al. [10], on the other hand compared symptoms of fecal incontinence between women with a minor (Grade 3a/3b) tear, with those with a major (Grade 3c/4) tear, showing that major tears were associated with higher risk of liquid fecal incontinence and lower anal canal pressures.

A disadvantage with ultrasound is the difficulty to distinguish between scar tissue and a residual defect. This can explain why few studies find strong correlations between symptoms of fecal incontinence and ultrasound defects. What looks like a defect might be a well repaired but scarred EAS, with normal function. Therefore, we aim to re-evaluate the patients in a successive control after one additional year, to assess if individuals with or without any persistent sphincter defects will show significant improvement or decline in fecal incontinence symptoms and sonographic findings. Voyvodic et al. [11] performed EAUS of more than 300 patients and found no correlation between the presence and extent of EAS defects found and the severity of fecal symptoms. Karoui [16] performed EAUS on 335 incontinent patients, 115 continent patients and 18 asymptomatic female volunteers. Anal sphincter defects were found in 43% of continent patients and 22% in asymptomatic volunteers.

Mahoney et al. [17], studied 500 patients sutured for a 3rd degree tear and showed that most women were asymptomatic or had minor symptoms. Full thickness IAS defects, but neither the presence nor extent of EAS defects were associated with functional impairment. However, symptoms of fecal incontinence may occur later in life and not always immediately after OASIS [17].

## Conclusion

In conclusion, correlations between the original grade of OASIS, symptoms of anal incontinence and residual defects as measured by two ultrasound modalities 6 months post-partum were found. Incontinence to gas and incontinence to liquid, correlated with specific anatomical defects, of which measurements by TPUS had similar or higher correlations than EAUS.

## Data Availability

The datasets generated during and/or analysed during the current study are available from the corresponding author on reasonable request
